# Biomechanical Comparison of Different Stabilization Constructs for Unstable Posterior Wall Fractures of Acetabulum. A Cadaveric Study

**DOI:** 10.1371/journal.pone.0082993

**Published:** 2013-12-31

**Authors:** Yuntong Zhang, Yang Tang, Panfeng Wang, Xue Zhao, Shuogui Xu, Chuncai Zhang

**Affiliations:** Department of Orthopaedics, Changhai Hospital, Second Military Medical University, Shanghai, China; Delft University of Technology (TUDelft), The Netherlands

## Abstract

**Purpose:**

Operative treatment of unstable posterior wall fractures of acetabulum has been widely recommended. This laboratory study was undertaken to evaluate static fixation strength of three common fixation constructs: interfragmentary screws alone, in combination with conventional reconstruction plate, or locking reconstruction plate.

**Methods:**

Six formalin-preserved cadaveric pelvises were used for this investigation. A posterior wall fracture was created along an arc of 40–90 degree about the acetabular rim. Three groups of different fixation constructs (two interfragmentary screws alone; two interfragmentary screws and a conventional reconstruction plate; two interfragmentary screws and a locking reconstruction) were compared. Pelvises were axial loaded with six cycles of 1500 N. Dislocation of superior and inferior fracture site was analysed with a multidirectional ultrasonic measuring system. Results: No statistically significant difference was found at each of the superior and inferior fracture sites between the three types of fixation. In each group, the vector dislocation at superior fracture site was significantly larger than inferior one.

**Conclusions:**

All those three described fixation constructs can provide sufficient stability for posterior acetabular fractures and allow early mobilization under experimental conditions. Higher posterior acetabular fracture line, transecting the weight-bearing surface, may indicate a substantial increase in instability, and need more stable pattern of fixation.

## Introduction

Fracture of the posterior wall is the most common acetabular fracture [1], [2]. They accounted for nearly 47% of the total acetabular fractures in the study by Letournel and Judet [2]. Operative treatment of these fractures with an unstable hip or when a large part of the posterior wall is involved has been widely recommended for anatomical reduction and rigid fixation [1]–[4]. However, Operative treatment of these fractures has produced varying results by different authors. It has generally been perceived that isolated fractures of the posterior wall have a good outcome [2], [4], but recent reviews have shown that 21% to 32% of patients have poor results [5]–[7]. Furthermore, only 82% of patients had a good to excellent result despite perfect reduction in 94% [2], and there is up to a 30% failure rate within one year after fixation of posterior wall fractures, even when surgically treated by experienced orthopaedic traumatologists [1]. As results, the gap or step in articular surface would induce the development of osteoarthritis and degeneration [2]–[3]. Moreover, redislocation is another severe complication of failure fixation [3]–[6], [9].

Although the failures are multifactorial, instable fixation method with followed premature mobilization is associated with poorer outcome [8], [10], [11]. It is clear that early postoperative rehabilitation training is beneficial to joint function recovery [2]–[8]. Therefore, beside anatomic reduction, a rigid fracture fixation implant, which allows early mobilization and prevents secondary displacement with a need for subsequent hip arthroplasty, seems to be of further importance in treatment of those fractures. A variety of fixation methods have been described for posterior acetabular wall fracture [1], [3]–[7], [9]–[11]. Generally, surgical fixation of posterior wall acetabular fractures is accomplished with two or three interfragmentary screws alone [9], [12] or in combination with a buttress plate [8], [13]. Several investigators have attempted to examine the contact area and load distribution of intact, fractured, and repaired cadaveric posterior acetabular wall with different fixation methods [3], [8], [11], . Others evaluate the stability of different fixation types for anterior wall, transverse, both-column, or T-type acetabular fractures [15]–[20]. But it is surprising that few biomechanical studies have been done to identify optimum technique of fixation for posterior wall fracture. Whether it is rigid enough by using screws fixation alone for posterior wall fracture? Is it necessary to combine with a buttress plate? How much can the additional plate contribute to the fixation stability? Can the locking plate provide more stability than conventional construct for the fractures? The aim of this laboratory study was therefore undertaken to evaluate static fixation strength of three common fixation constructs: interfragmentary screws alone, in combination with conventional reconstruction plate, or locking reconstruction plate.

## Materials and Methods

### Fracture model

The Committee on Ethics of Biomedicine Research, Second Military Medical University approved the study. Written informed consent was obtained from the donor or the next of kin for use of this sample in medical research.

Six complete human pelvis which were including the forth lumbar vertebra and proximal 1/3 femoral shaft were obtained from the formalin-preserved cadavers of six adult males, without known metabolic bone disease or tumors. The specimens were harvested with a layer of periosteum and muscle insertions intact and with all ligaments and capsules of both hip joints intact. Mean age was 62 years (ranged of 45–76 years) at the time of death. The trabecular appearance and bony quality were examined and bone abnormalities were ruled out by a standard anteroposterior X-ray. Soft tissue was removed, except ligaments of the sacro-iliac joint and pubic symphysis.

**Figure 1:**
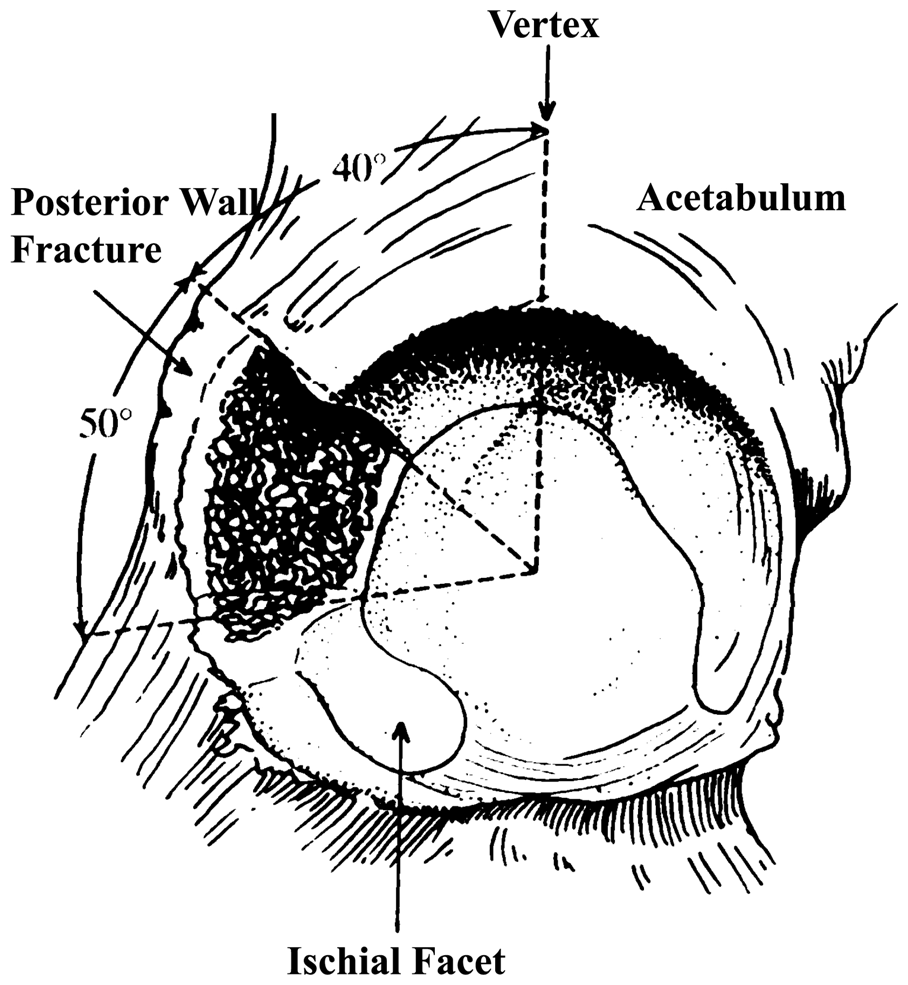
Illustration of the simulated posterior wall fracture of the acetabulum. The fracture began from 40 degree posterior to the acetabular vertex and extended another 50 degrees. The simulated fracture created a defect of the entire width of the articular surface of the posterior wall with this 50 degrees arc. The inferior portion of the articular surface of the posterior wall (the ischial facet) remained intact.

The simulated fracture line of posterior wall was designed and painted on the basis of the work of Olson SA et al [8], [11].The fracture began from 40 degree posterior to the acetabular vertex and extended another 50 degrees with the entire width of the articular surface of the posterior acetabular wall (Fig. 1). The fragment include more than 50% of the surface area of the posterior acetabular wall.

### Test set up

The specimen was fixed in the position of double-limb stance as previously described by Sawaguchi et al [21] and our former study [14]. The specimen was placed in a specific neutral position defined with the iliac wings level (coplanar in the horizontal plane) and with the plane formed by the anterior superior iliac spine and the pubic symphysis aligned vertically [11]. Osteotome was used to eliminate redundant intervertebral disk and other soft tissue and we made a platform for load test using selfcuring denture acrylic on the top of the forth lumbar vertebra. The proximal aspect of each femoral shaft was anchored into an aluminum tube with polymethylmethacrylate and screws fixation. The femoral shafts were placed in 15 degrees of adduction relative to the pelvis in frontal plane and oriented in 5 degrees of internal rotation.

### Tested groups

Three of specimens were tested in following order:

1. The simulated fracture of the posterior wall was created according to the painted line described above. Oscillating saw was used to create a realistic fracture in the articular surface. The fragment then was anatomically reduced and fixed with two 4.0 mm cancellous screws (Weigao Orthopedic Device Co., Limited, Shandong, China).
2. After that, the posterior wall fragment was buttressed by a standard 7-hole 3.5 mm conventional reconstruction plate and 4 cortical screws (Weigao Orthopedic Device Co., Limited, Shandong, China).

The other three specimens were tested in the same procedures, but standard 7-hole 3.5 mm locking reconstruction plate and 4 locking cortical screws (Weigao Orthopedic Device Co., Limited, Shandong, China) were used instead. These three conditions will subsequently be referred to as screws, conventional reconstruction plate with screws (CPS), and locking reconstruction plate with screws (LPS). There are 6 specimens in screws group, 3 in CPS group, and 3 in LPS group.

**Figure 2:**
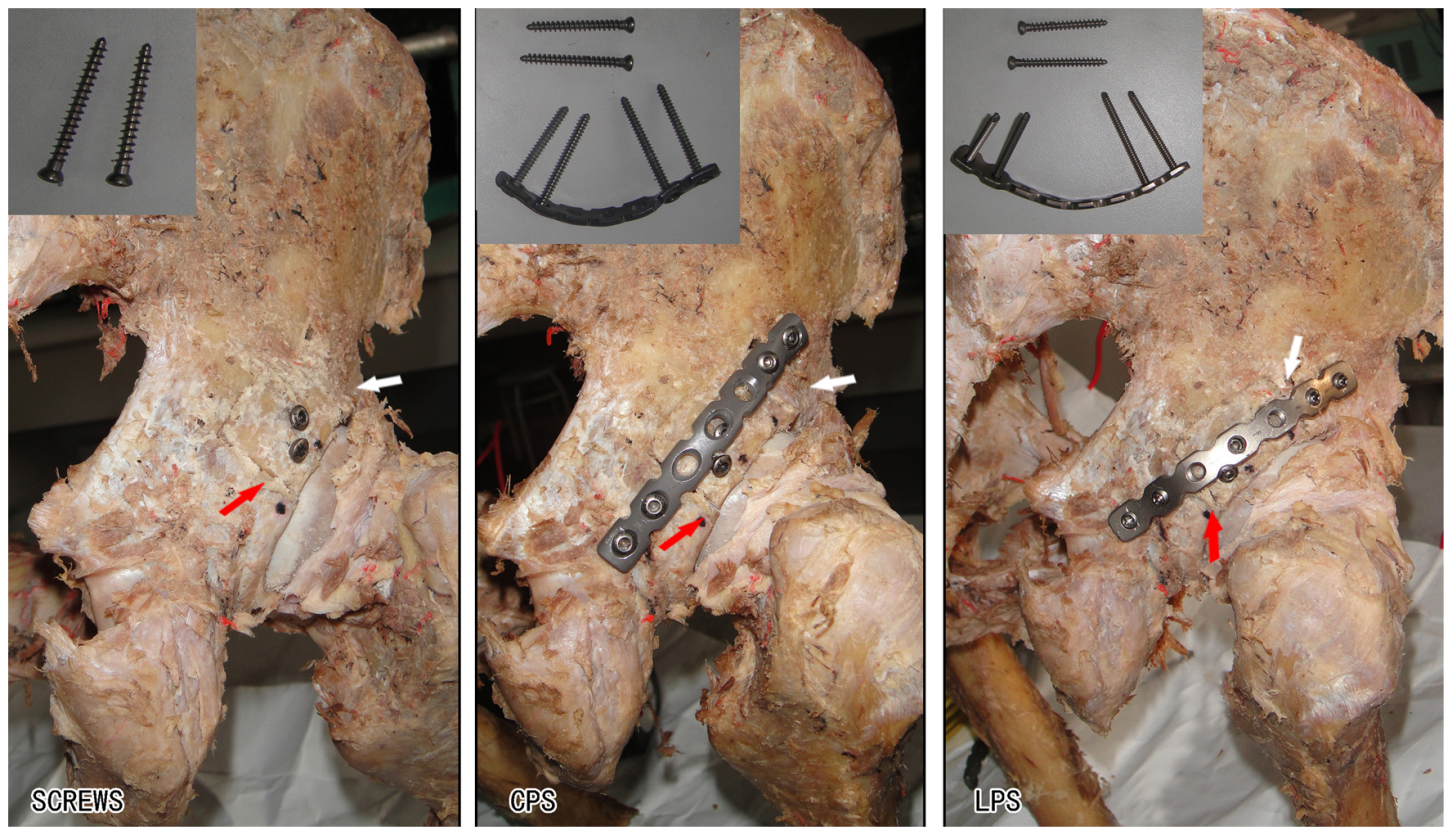
Groups of different fixation constructs tested. SCREWS = two 4.0 mm cancellous screws. CPS = two 4.0 mm cancellous screws and a standard 7-hole 3.5 mm conventional reconstruction plate with 4 cortical screws; LPS = two 4.0 mm cancellous screws and a standard 7-hole 3.5 mm locking reconstruction plate with 4 locking cortical screws; white arrow = superior fracture line; red arrow = inferior fracture line.

All procedures were performed by one of us (Shuo-gui Xu), an experienced surgeon. All reductions were anatomical. There were small gaps in the interface between the fragment and intact bone that were less than 1 mm (Fig. 2).

### Testing protocol

**Figure 3:**
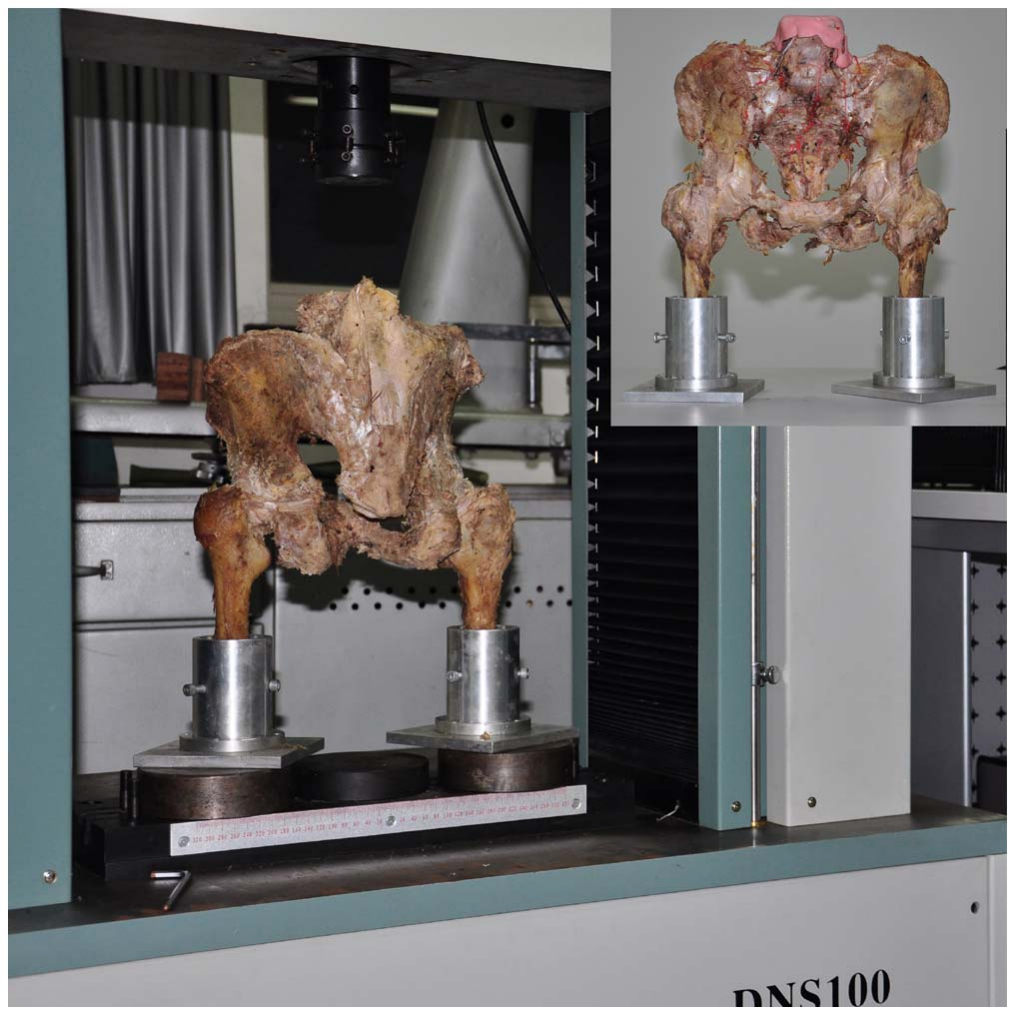
The load cell and jig used to position the pelvis and femur. Test set up: The pelvis is mounted in the up right position, simulating a double-limb stance, free movable in all three planes. The axial load is applied through the forth lumbar vertebra. The fracture dislocations, under axial loading were analysed. Insert at top right = Test set up in anterior-posterior view.

The mounting hardware and pelvis were attached superiorly to a CSS-44000 electromechanical universal testing machine (Changchun Research Institute for Testing Machine Co., Ltd, Changchun, China) as shown in Fig. 3. All 6 pelves were axially loaded with six cycles up to 1500 N (10 mm per minute) for each three condition, simulating a static two times the body weight of a 70 kg person. The first cycle was used to achieve elasticity of the setup, and the following five cycles were used for the measurements. The break-off criteria was defined as fracture displacement >2 mm or an implant- or pelvic- breakage. Motion at the fracture site in three orthogonal directions, and the overall stiffness of the construct, were recorded simultaneously.

### Measurement System

For the real-time analysis of fracture dislocation, an ultrasonic measuring device (CM-S70P; Software Win Biomechanics v0.2.6; Zebris, Isny, Germany) with 2 independent sensor pairs were performed in the superior and inferior fracture line to determine the maximal displacement in the region of interest. For this measuring method, a broad experience and knowledge of data analysing exists [17], [19], [22], [23].

The sensor pairs consisted of an ultrasonic microphone and an ultrasonic reader allowing for data collection of three different motion parameters. The sensor pairs were applied to the posterosuperior portion of remaining acetabular wall and the fractured fragment (superior sensor pair; measurement of superior fracture line site dislocation of the fragment) as well as to the inferior portion of remaining acetabular wall and the fractured fragment (inferior sensor pair; measurement of inferior fracture line site dislocation of the fragment). The tracking system recorded a full set of kinematic data in the x-, y- and z-axis (over 6000 values per pelvis), with a rate of 5 Hz and an accuracy of 2.5% of the measured distance. The z-axis represents displacement appropriately in the anterior-posterior direction, the x-axis in the anterior-posterior direction and the y-axis in the vertical direction. DELTA values (maximum–minimum) of all three translation-parameters were calculated using the mean plateau values of each load and unload per cycle. Vector calculation of the three translation axes was used to evaluate the real fracture dislocation direction and amount.

### Statistical methods

All data are presented as mean ± standard deviation. Data of vector dislocation of different experimental groups were analyzed by Scheffe' post hoc test and one-way ANOVA. A confidence level of 95% was considered to indicate significant differences. For statistical analysis SPSS software package, version 18.0 for Windows (SPSS Inc., Chicago, IL, USA) and the statistical outlier test by Grubbs was used.

## Results

**Figure 4:**
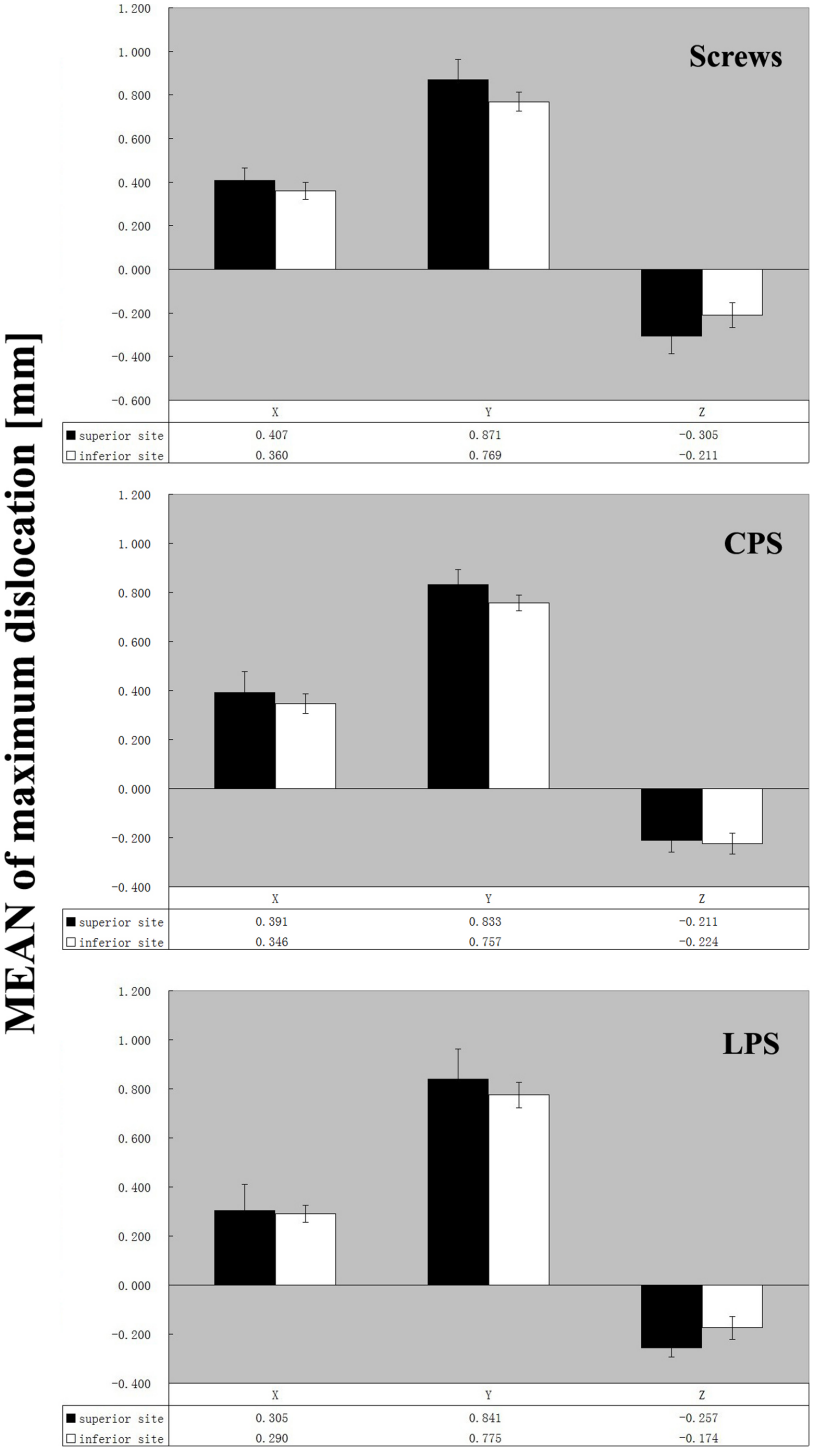
Mean fracture translation in all three axes for each construct. Dark bars = at superior fracture site (marked with a white arrow in Fig. 2); bright bars = at superior fracture site (marked with a red arrow in Fig. 2). n = 30 for Screws group, 15 for CPS and LPS group.

Independent of the fixation construct (plates or screws), the motion pattern in the superior and inferior fracture line was similar in all three groups, as shown for the three translation axes (Fig. 4). All vector displacements were below the clinically tolerable maximum value of 2 mm. Therefore each tested fixation technique was able to stabilize fractures of the cadaveric pelvises up to a vertical load of 1500 N.

**Figure 5:**
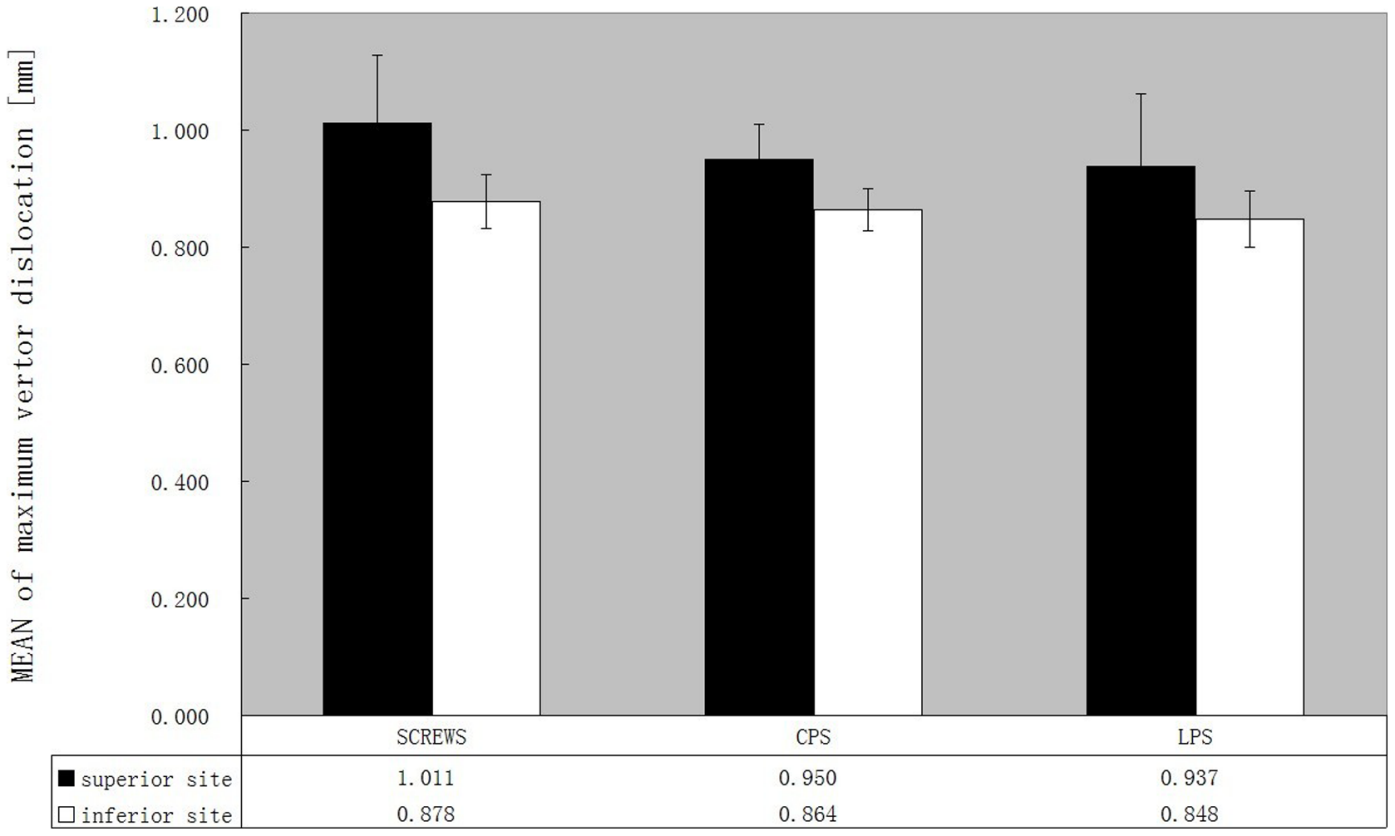
Mean vectors of fracture dislocations for the different constructs. Dark bars = at superior fracture site (marked with a white arrow in Fig. 2); bright bars = at superior fracture site (marked with a red arrow in Fig. 2). n = 30 for Screws group, 15 for CPS and LPS group.

In evaluating the superior fracture line, the screws group made numerically the largest difference in the degree of dislocation, although it did not reach statistical significance. Comparatively, the dislocations of three constructs were approximately Equivalent at the inferior fracture site. The additional buttress plate (conventional or locking) prevented displacement more sufficiently than using screws alone. However, no statistically significant difference was found at each of the superior and inferior fracture sites between the three types of fixation (Fig. 5, Table 1). In each group, the vector dislocation at superior fracture site was significantly larger than inferior one (Table 2).

**Table 1:** the mean vector dislocation for three tested constructs.

	Superior (mm) (Mean ± SD)	Inferior (mm) (Mean ± SD)	Difference^2^
Screws (n = 30)	1.011±0.12	0.878±0.05	t = 5.76, p<0.01
CPS (n = 15)	0.950±0.06	0.864±0.04	t = 4.56, p<0.01
LPS (n = 15)	0.937±0.12	0.848±0.05	t = 2.51, p = 0.018
Difference^1^	F = 2.893, p = 0.064	F = 2.154, p = 0.125	

^1^ , based on analysis of variance.

^2^ , based on group t-test.

Alpha = 0.05.

**Table 2:** Significances for the Relative Fixation stiffness of Different Constructs.

Superior fracture site	Screws	CPS	LPS
Screws	-	0.217	0.114
CPS	0.217	-	0.955
LPS	0.114	.0955	

Inferior fracture site	Screws	CPS	LPS
Screws	-	0.637	0.130
CPS	0.637	-	0.636
LPS	0.130	0.636	-

All data are given as p-values. Multiple testing using the post hoc scheffe test. A confidence level of 95% was considered to indicate significance accordingly (alpha = 0.05).

## Discussion

Open anatomic reduction and rigid fixation is the standard operative procedure for the treatment of displaced posterior wall fractures of the acetabulum to allow early mobilisation and prevent secondary displacement with a need for subsequent hip arthroplasty [2]. Restoration of normal anatomy should preserve function of the joint and thereby prevent later degenerative changes that culminate in osteoarthrosis [1], [7]. Howerer, post-traumatic osteoarthrosis of the hip joint has been reported in association with as many as 20% of fractures of posterior wall of acetabulum after treatment with open reduction and internal fixation [2], [7]. Rowe and Lowell [24] observed that posterior instability after repair of these fractures is an additional predictor of osteroarthrotic sequelae. In recent long-term clinical study [9], radiographic and clinical outcomes were directly correlated with the degree of reduction achieved. If displacement of 2 mm or less was present after surgery, both radiographic and functional outcomes were excellent or good in the vast majority of the patients. In cases of residual displacement of 2 mm or greater only half of the patients showed satisfactory results at the final follow-up. Olson SA et al [3], [8] test the mechanics of load transmission across the hip after a fracture of posterior wall of acetabulum and find an increase in contact area, maximum pressure, and contact force in weight-bearing portion of acetabulum. And they found the pattern of loading did not restore after anatomical reduction and fixation of the fracture with a plate and screws. Then they used calcium phosphate cement as an adjunct to internal fixation for posterior wall acetabular fracture and found it resulted in a partial restoration of joint loading parameters toward the intact state [11].

One explanation for the similar findings in those studies is the step-off or gap at the fracture site due to instability of fixation method, in other words improving stability of fixation could decrease the movement of fractured fragment and then results in restoration back to levels similar to the normal condition in contact area and load distribution.

Failure to achieve and maintain accurate reduction has been seen as the prime cause of poor outcomes [4], [7], [25]. To verify clinical knowledge, a few biomechanical studies have evaluated the stabilisation methods of acetabular fractures. Sawaguchi et al [21] repaired the anterior column with a plate or lag screw, and the posterior column was fixed with one of three different plates. No differences were reported between the various modalities. Mehin R et al [26] suggested that the locking plate construct is as strong as the conventional plate plus interfragmentary lag screw construct for fixing transverse acetabular fractures. Simonian PT et al [18] evaluated the stability of different types of fixation for the T-type acetabular fracture and find the differences in displacements measured were not statistically significant. Schopfer A et al [27] found that lag-screw fixation plus neutralisation plating provided greater stability in posterior column model at 60°of hip flexion. However, to our knowledge, only one previously published study in 1994 [28] has provided information about the stability of fixation of posterior acetabular wall fractures. The authors simulated concentric comminuted and transverse comminuted posterior wall fracture of acetabulum separately. The stiffness of a reconstruction plate and screws was observed significantly higher than that with screws alone. Further, no biomechanical evaluation of the stiffness of fixation with locking plate was provided for posterior acetabular wall fracture.

Reconstruction plates, buttressing the posterior wall, in conjunction with interfragmentary screws are the most common fixation method for posterior acetabular wall fracture. In our current study, the conventional plate and interfragmentary screws facilitate a rigid fracture fixation at 1500N load with a mean displacement <1 mm. the result are consistent with the recent clinical observation [23]. But differ with the previous study [13], the additional buttress reconstruction plate did not significant increase the fracture fixation strength compared to screws fixation alone in our study. The difference, as we conclude, may attribute to two causes as followed: first, the stiffness of fixation with a reconstruction plate and screws measured in the previous study was 8205 N per millimeter, which was significantly higher than that achieved with screws alone (2083 N). But the stiffness at a level of 1500 N, which is roughly two times the body weight of a 70 kg person, was not compared. The authors chose a load of 1500 N, which simulated a load of hip joint in a single-leg stance position. This is approximately maximum load that might be anticipated during rehabitiation and allow sitting up on bed and partially weight bearing with crutches in the early time after operation.

Second, accordingly, the posterior wall fracture was created in a semicircular pattern which may affect the load distribution. In addition, the simulated fracture fixed by screws alone was created to transverse comminution, and each fragment was fixed only by one screw. Comparatively, our fracture pattern and fixation method was more similar to clinical data [1], [10], [12] and recent biomechanical studies [8], [11], [14].

Interfragmentary screws have been advocated as the best initial stabilisation of acetabular fractures [12], [23]. Gun II et al [29] use screws alone for treatment of single fragmented or moderately comminuted posterior wall fracture of the acetabulum. Excellent to good results were achieved for 14 (93%) of 15 patients. Although the use of a reconstruction plate and screws appear to be stronger than using screws alone, the latter construct still may be safe in vivo. In the current experiment, after six cycles of cyclic loading, there is still less than 1 mm of gap at the fracture site. We suggest that single or large fragmented posterior acetabular wall fracture can be safely fixed by two or more interfragmentary screws. Further advantages of the screw fixation constructs compared with the common plate fixations are the possibility of minimal invasive approach and placement if an anatomic closed reduction can be achieved.

With a population getting older, injuries of the aged will further increase, as already observed over the last 15 years in an analysis of 1266 cases treated by the German Multicentre Study Group (DAO/DGU) [30], [31]. Restrictions of treatment have to be considered in this group of patients. Locking plates are becoming popular in orthopedic trauma management for postulated increased stability of fracture fixation especially in osteopenic bones. Several biomechanical studies indicated the locking plate can provide more stability than conventional construct for acetabular fracture [31], [32]. Surprisingly, the stiffness of fracture fixed by LPS was higher than NPS, but did not reach significance in our study. The results, as we conclude, might attribute to the use of formalin-preserved cadavers, which may not represent osteoporotic bone quality. Besides the increased strength, the advantages of locking plate are various. Locking plates do not depend on plate-bone contact and friction to achieve stability. Fracture fixation with a conventional plate relies on the compressive force provided by the screw head to the plate and the friction coefficient between plate and bone [33]. Insufficient contouring of conventional plates lead to insufficient compressive force from the screw head to the plate or insufficient friction between the plate and the bone will result in compromise of stability across the fracture site, and potential poor stability of fixation. Perfect contouring is much easier in cadaveric study than that in vivo. Therefore, intraoperatively, the locking plate is less likely to act as a deforming construct disrupting fracture reduction, but provide more potential stiffness. Furthermore, as reported, an additional monocortical screws via the locking plate can be used to enhance the construct strength without risk of articular violation and prolonged use of intraoperative fluoroscopy to assure extraarticular placement of lag screws [34].

It is noteworthy that, though all fixation implants facilitated a rigid fracture fixation with a maximum dislocation <2 mm, the displacement of superior fracture line was larger than the inferior one. That may indicated that if the fracture line is the higher in acetabular dome, transecting the weight-bearing surface, a substantial increase in instability may be encountered, and more stable pattern of fixation is essential. The simulated fracture in our study began from 40 degrees posterior to the acetabular vertex and extended another 50 degrees of the posterior acetabular wall, which was considered the most common pattern of posterior wall fracture [8], [11]. However, the location of posterior acetabular wall fracture varies according to the position and flexion angle of the hip [35]. Hence the fixation stability of lower degree fracture such as 15 to 60 degree of the posterior acetabular wall, which may due to the direct trauma on the anterior aspect of flexed knee with the hip flexed to 60 degrees and in slight or no abduction [31], will be required for a definitive assessment in further biomechanical experiments and clinical studies.

As common in biomechanical studies, the current study is limited by the use of formalin-treated specimens. Fresh specimens or clinical trials would enhance subsequent studies. In addition, the absence of active muscular deforming forces provides an obvious potential source of an experimental bias. Therefore the results of this study have to be interpreted with caution and absolute values of fixation strength cannot be extrapolated to the clinical situation. Meanwhile, it should be noteworthy that static standing is not the activity generating maximum acetabular contact pressure at posterior wall surface during weight-bearing exercise. As reported in recent study [36],the contact pressures at the posterior horn are much higher during standing up from a chair or sitting down activities. But the authors think that this study supplies biomechanical guidelines for selecting configurations that can maintain the stabilization for posterior wall fracture of acetabulum and allow early non- or partial weight bearing mobilization after operation. Therefore, we have evaluated stability only in standing position with axial loading. The findings cannot be applicable to sitting and sit to stand position.

On the other hand, for eliminating variables as possible and focusing on pure comparison of the different fixation configurations, comminution and impaction of the articular surface were not part of the model tested in our study. In spite of that, according to our resulting data and previous records [24], it can be concluded that large-size fragment of comminuted fracture of posterior wall of aetabulum can be stably fixed by two or more interfragmentary screws, but in case of small fragments, only one screw is insufficient and an additional buttress plate is recommended for enough stabilization.
